# Dual RF Input Envelope Tracking Power Amplifier with Enhanced Load Modulation for Power–Efficiency–Linearity Trade-Off

**DOI:** 10.3390/s26123897

**Published:** 2026-06-19

**Authors:** Marco Badii, Giovanni Lasagni, Monica Righini, Giovanni Collodi, Stefano Maddio, Alessandro Cidronali

**Affiliations:** Department of Information Engineering, University of Florence, Via di Santa Marta, 3, 50139 Florence, Italy; giovanni.lasagni@unifi.it (G.L.); monica.righini@unifi.it (M.R.); giovanni.collodi@unifi.it (G.C.); stefano.maddio@unifi.it (S.M.)

**Keywords:** envelope tracking, load modulation, GaN power amplifier, dual RF input PA

## Abstract

In this paper, we present an optimized driving strategy for a dual RF input envelope tracking power amplifier (ET PA) exploiting load modulation. The dual-input architecture enables dynamic load modulation (LM), allowing real-time adjustment of the load impedance to enhance performance over the signal dynamics typical of digital modulation schemes. The proposed approach considers a GaN HEMT-based LM-ET PA characterized under pulsed excitation across multiple amplitude and phase conditions of the load modulation control. Optimizing the control parameters yields a suitable shaping function that extends conventional ET supply modulation to include amplitude and phase control of the auxiliary amplifier, thereby improving the efficiency, output power, and linearity of the main amplifier. Experimental data demonstrate that the proposed dual RF input GaN-based LM-ET PA at 3.6 GHz outperforms a conventional ET PA in both efficiency and linearity when tested with high peak-to-average ratio (PAPR) signals.

## 1. Introduction

Modern mobile communication systems increasingly rely on wideband signals with high peak-to-average power ratio (PAPR) to support high data-rate transmission. Representative examples include LTE/LTE-Advanced, 5G New Radio (NR), Wi-Fi 6/7 systems based on IEEE 802.11ax/be, and digital broadcasting standards such as DVB-T/T2, where OFDM- or OFDMA-based waveforms produce large envelope fluctuations [1,2,3,4]. Under these conditions, conventional fixed-supply power amplifiers (PAs) must operate with significant output back-off (OBO) to meet linearity and spectral-mask requirements, resulting in a consequent degradation of average efficiency.

Several efficiency-enhancement techniques have been developed to address this issue [4,5,6,7]. Envelope tracking (ET) is one of the most effective solutions for signals with large envelope dynamics, as it adapts the drain supply voltage to the instantaneous signal envelope and therefore allows the PA to operate closer to high-efficiency conditions over a broad power range [8,9]. However, since conventional ET primarily acts through supply modulation while the RF load remains fixed, reducing the drain voltage may also limit the achievable delivered power. As a result, ET design involves a trade-off among average efficiency, output power, and linearity, which also depends on the signal probability distribution [10].

A complementary approach to efficiency enhancement is dynamic load modulation (LM). The most established example is the Doherty PA, where active load modulation is exploited to improve back-off efficiency [11]. Doherty architectures are attractive because of their relatively simple RF implementation, but their performance is affected by the impedance inverter, the interaction between carrier and peaking amplifiers, and the difficulty of simultaneously achieving wide bandwidth, high efficiency, and good linearity. More recently, load-modulated balanced amplifiers (LMBAs) have extended the load-modulation concept toward improved broadband operation by adding an auxiliary control amplifier to a balanced PA structure [12]. Further developments have combined load and supply modulation, as in supply-modulated LMBAs [13], while dual-RF-input PAs have also been investigated for Doherty and multi-mode architectures [14,15]. These solutions show that additional RF or bias control degrees of freedom can enlarge the accessible performance space, but they also require the synthesis of suitable control laws rather than the optimization of isolated operating points.

Within this framework, the load-modulated envelope-tracking (LM-ET) approach considered in this work combines the advantages of ET and RF load modulation. The drain supply voltage provides the conventional ET efficiency-control mechanism, while the auxiliary RF input dynamically modifies the effective load seen by the main PA. This coordinated control offers an additional degree of freedom to trade output power, efficiency, gain behavior, and linearity under high-PAPR operation.

The LM-ET architecture considered here was introduced and experimentally validated in [16], where the focus was on the analytical interpretation of the load-modulation mechanism and on static and two-tone proof-of-concept measurements. The present paper does not introduce a different RF hardware architecture. Its contribution is instead methodological: it addresses the problem of defining the dynamic driving strategy required to operate the architecture with envelope-varying signals. To this aim, pulsed measurements are performed on a GaN HEMT-based dual-RF-input LM-ET PA to reduce thermal and trapping effects that are particularly relevant in GaN technologies [17]. The resulting multidimensional experimental dataset is then used to synthesize Pareto-optimal shaping functions for the Main’s drain supply voltage and for the amplitude and phase of the Auxiliary’s RF input.

The main objective is therefore to transform the experimentally characterized LM-ET operating space into physically admissible control trajectories. These trajectories are selected according to Pareto-optimal trade-offs among output power, efficiency, gain flatness, and linearity-related metrics, with the aim of assessing how the additional load-modulation degree of freedom can improve or reshape the performance attainable with respect to a conventional ET benchmark.

The remainder of the paper is organized as follows. Section 2 describes the dual-RF-input LM-ET PA architecture and recalls the reference power-scaling mechanism that motivates the use of load modulation in combination with envelope tracking. Section 3 presents the experimental characterization setup and the measured multidimensional dataset. Section 4 introduces the Pareto-driven framework adopted to derive admissible shaping functions for the supply voltage and auxiliary-path controls. Section 5 analyzes representative Pareto-optimal shaping strategies and compares the resulting LM-ET performance trajectories with a conventional ET benchmark. Finally, Section 6 summarizes the main findings and outlines future developments toward fully dynamic wideband validation.

Table 1 summarizes the main literature context behind the efficiency-enhancement techniques discussed above. The selected works cover polar and supply-modulated PAs, envelope tracking, Doherty and active load-modulated architectures, dual-RF-input solutions, and measurement-based characterization approaches. Rather than ranking these contributions only by absolute performance, the table is intended to clarify the positioning of the present work: starting from the LM-ET hardware concept, this paper focuses on the Pareto-based synthesis of admissible supply-voltage, auxiliary-amplitude, and auxiliary-phase shaping laws from measured multidimensional data.

## 2. The Dual RF Input Envelope Tracking PA

### 2.1. The Architecture

**Figure 1:**
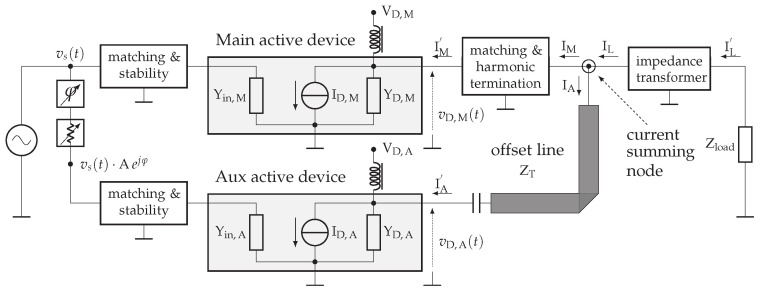
Dual RF input ET PA schematic architecture.

The dual-RF-input ET PA architecture is depicted in Figure 1. It comprises two RF power devices driven by a pair of coherent input signals, with their outputs combined at a common summing node. The combination is achieved via an offset transmission line, which enables controlled load modulation of the main PA through the auxiliary path and prevents loading of the auxiliary path onto the main PA under low-signal conditions. For low levels of auxiliary injection, the main PA is effectively terminated at its optimum load impedance, as determined by signal statistics criteria [10]. Such an optimal load condition results from the combined action of the impedance-transformation network and the output-matching.

The main amplifier is biased to operate under optimal envelope tracking conditions, whereas the auxiliary amplifier operates in class-B or Class-C (i.e., with negligible quiescent current in the absence of RF excitation). Compared with conventional ET architectures, the inclusion of an auxiliary RF input—characterized by independently controllable amplitude and phase—introduces an additional degree of freedom, enabling continuous, dynamic control of the load impedance presented to the main ET PA.

Unlike conventional ET PA configurations, the proposed architecture enforces explicit synchronization between RF-domain load modulation and the low-frequency supply modulation inherent in ET operation. This coordinated control framework enables precise shaping of the load trajectory experienced by the main device, resulting in enhanced output power capability and improved linearity under back-off conditions, while preserving the efficiency advantages associated with envelope tracking.

Figure 1 shows the architecture of the proposed Dual Input Envelope Tracking Device. It is based on a pair of 8 W GaN-HEMT devices capable of operating up to 6 GHz [18]. The two PAs operate in Class-AB and Class-C, respectively, for the main and auxiliary modules. It is worth noting that the present work does not focus on the detailed design of the individual PA blocks, which were already introduced and experimentally validated in [16]. The matching and harmonic termination and stabilization networks shown in Figure 1 follow conventional design criteria based on the device manufacturer recommendations, small-signal stability requirements, and the desired fundamental and harmonic terminations. In particular, the output impedance transformation network of the main branch is designed to present the optimum load impedance to the main PA when the auxiliary PA is inactive, thus defining the reference ET operating condition. The distinctive element of the proposed topology is instead the offset line connecting the main and auxiliary paths to the summing node. This line is designed to limit undesired interaction between the main and auxiliary amplifiers at low power levels, when the auxiliary contribution should not significantly load the main PA. At the same time, it enables the auxiliary path to behave, at the summing node, as an effective controlled current source. This condition is essential to transform the auxiliary RF drive into a dynamic load-modulation mechanism acting on the main PA, while preserving a well-defined ET operating condition when load modulation is not required.

### 2.2. Reference Power Scaling of ET and LM-ET Operation

The purpose of this subsection is to recall the ideal power-scaling mechanism of conventional envelope tracking (ET) and load-modulated envelope tracking (LM-ET) operation. This discussion is not intended to provide a quantitative prediction of the measured prototype performance, which is affected by device nonlinearities, losses, finite harmonic terminations, and the actual auxiliary-path control. Rather, it provides a qualitative reference used in the following sections to interpret why the proposed dual-input LM-ET architecture can reach output-power trajectories that are not available to a conventional ET benchmark.

As discussed in [16], a simplified linear PA model can be used to highlight the different power scaling of the two operating principles. The drain current waveform is described by its peak value, $I_{D,M/A}^{\left(pk\right)}$, while its DC and fundamental components are represented through the Fourier coefficients $c_{M/A}^{\left(DC\right)}\left(\theta \right)$ and $c_{M/A}^{\left(1\right)}\left(\theta \right)$, where the subscripts *M* and *A* refer to the main and auxiliary amplifiers, respectively. Under ideal harmonic terminations, only the DC and fundamental components are considered in the power balance.

For the main PA, the optimum load resistance at a given drain supply voltage can be written as

(1) $$R_{L,\mathrm{opt}}=\frac{1}{c_{M}^{\left(1\right)}}\frac{V_{D}-V_{K}}{I_{D,M}^{\left(pk\right)}},$$

where $V_{D}$ is the main drain supply voltage, and $V_{K}$ is the transistor’s knee voltage. Equation (1) shows that, for a fixed current peak, the optimum load resistance decreases when the supply voltage is reduced. For example, in class-B operation, cM(1)=12 and cM(DC)=1π.

In a conventional ET PA, the supply voltage is reduced according to

(2) $$V_{D}=kV_{D,\max},\;0<k\leq 1.$$

Since the RF load is fixed, the PA cannot continuously track the optimum load condition given by (1). In the ideal reference case, both the voltage and current swings scale approximately with *k*, and the delivered output power therefore follows the quadratic trend

(3) $$\frac{P_{L}^{ET}}{P_{L,\max}}∼k^{2}.$$

The LM-ET architecture introduces an additional degree of freedom through the auxiliary RF path. The auxiliary amplifier injects current at the fundamental frequency that modifies the load seen by the main amplifier. The corresponding effective load can be written as

(4) $$R_{M}=R_{L}\left(1+\frac{I_{D,A}^{\left(1\right)}}{I_{D,M}^{\left(1\right)}}\right),$$

where $I_{D,M}^{\left(1\right)}$ and $I_{D,A}^{\left(1\right)}$ are the fundamental current components of the main and auxiliary amplifiers. By properly controlling the amplitude and phase of the auxiliary contribution, the effective load $R_{M}$ can be moved closer to the optimum value required by the instantaneous supply voltage.

Under ideal optimum load modulation, the main current swing does not need to decrease in proportion to the supply voltage. Instead, the current can remain close to its maximum value while the voltage swing scales with $kV_{D,\max}$. In this case, the delivered power follows the approximately linear trend

(5) $$\frac{P_{L}^{LM}}{P_{L,\max}}∼k.$$

**Figure 2:**
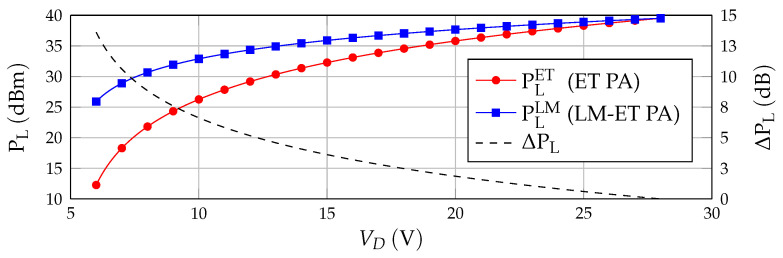
Theoretical ET PA and LM-ET PA model performances, evaluated for $V_{K}$ = 6 V, $V_{D,\max}$ = 28 V, $V_{D,A}$ = 12 V, $I_{D,\max}$ = 1.7 A, $\theta_{M}$ = 1.1 π and $\theta_{A}$ = 0.8 π. Adapted with permission from [16].

Equations (3) and (5) are the key results recalled here. They show that, when the drain supply is reduced, a conventional ET PA ideally loses output power with a quadratic dependence on *k*, whereas an LM-ET PA can ideally reduce the output power only linearly. Equivalently, for a halving of $V_{D}$, the LM-ET case exhibits an intrinsic power advantage of about 3 dB with respect to conventional ET operation. Both architectures reach the same maximum output power at $V_{D,\max}$, but they are expected to diverge at reduced supply voltages, as illustrated by the reference trends in Figure 2.

This ideal comparison is used in the remainder of the paper as an interpretative baseline. The experimental analysis does not attempt to verify (3) and (5) as exact closed-form predictions. Instead, the measured dataset is used to synthesize physically admissible shaping functions for $V_{D}$, auxiliary attenuation and auxiliary phase. The resulting LM-ET trajectories are then compared with a conventional ET benchmark to assess whether the additional load-modulation degree of freedom actually moves the measured amplifier behavior toward higher delivered power, improved iso-gain feature, and different efficiency–linearity trade-offs. In this sense, the theoretical trends summarized in this subsection provide the rationale for the benchmark comparison reported later in the paper.

## 3. Device Characterization and Measurement Dataset

**Figure 3:**
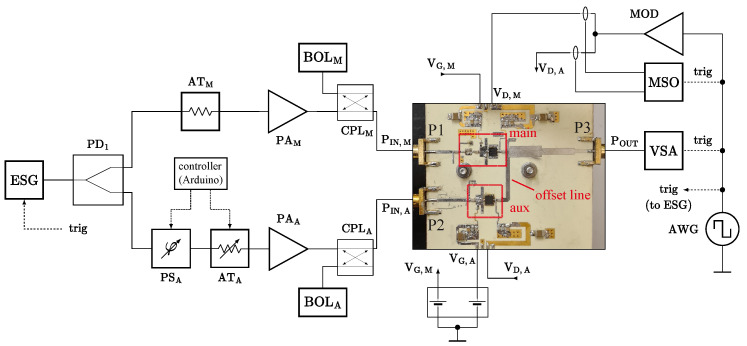
Photograph of the fabricated LM-ET PA prototype together with a schematic representation of the experimental characterization setup. Adapted with permission from [16].

The proposed LM-ET PA was experimentally characterized using the setup schematically shown in Figure 3, where a photograph of the fabricated prototype is complemented by the input power splitter and the amplitude- and phase-control elements in the auxiliary branch. The bias supply was pulsed for 9 μs, and drain currents were measured using current probes. The measurement arrangement was designed to systematically explore the complete LM-ET operating space under controlled and repeatable conditions. Since the proposed architecture depends simultaneously on the input-envelope level, the main drain supply voltage, and the auxiliary-path amplitude and phase controls, non-modulated RF excitations with different amplitudes were used to map the multidimensional operating domain in a waveform-independent manner. This map is later used to reconstruct the performance associated with different envelope-dependent shaping functions and signal statistics. Pulsed measurements were adopted to minimize self-heating and drain-current lag effects, which are particularly relevant in GaN HEMT devices [19]. The resulting dataset therefore provides a quasi-static description of the intrinsic LM-ET behavior, while high-PAPR operation is introduced in the subsequent analysis through statistical weighting of the measured operating points and offline reconstruction of the corresponding LM-ET trajectories.

**Figure 4:**
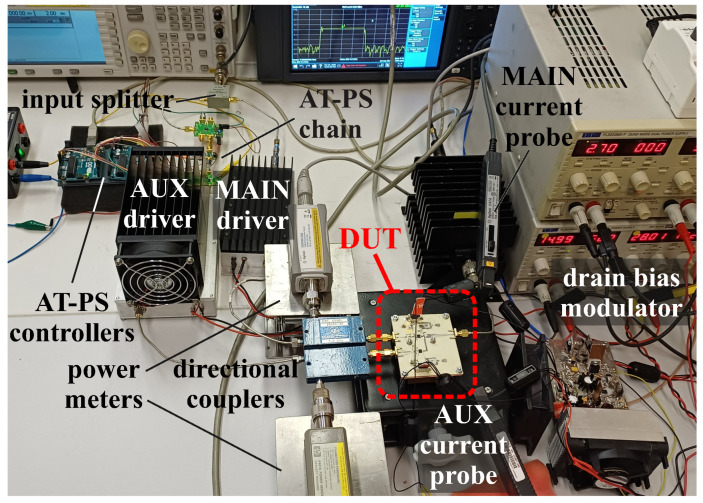
Photograph of the complete measurement setup.

Figure 4 shows the laboratory setup adopted for the prototype characterization. The figure highlights the driver amplifiers, the current probes, and the input couplers used for prototype input power measurements, while the amplitude and phase control units are visible behind the driver stages.

In the selected bias condition of $V_{D}=28\;V$, $I_{D,M}^{\left(DC\right)}=52\;mA$, and $I_{D,A}^{\left(DC\right)}=0\;mA$, the prototype exhibits a 3.6 GHz small-signal power gain approaching 17 dB, with input return loss lower than −15 dB at both ports.

**Figure 5:**
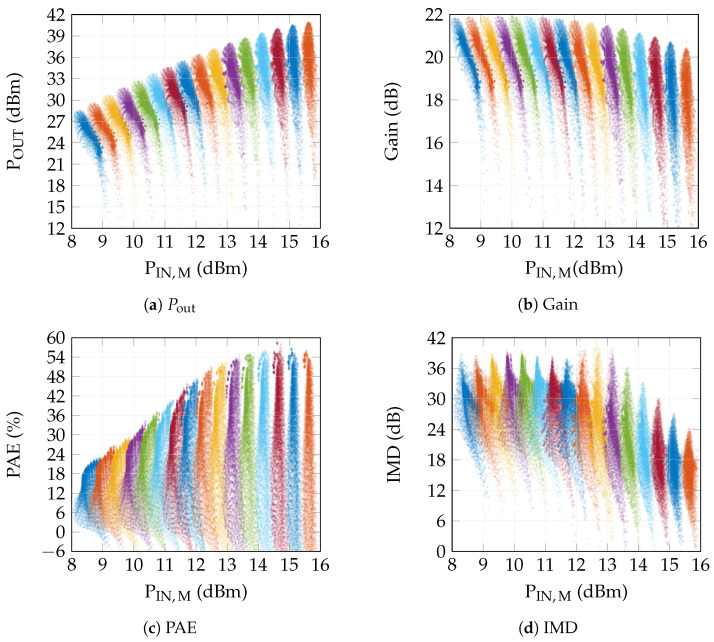
Measured clouds, obtained at different nominal input power settings, of the performance metrics: (**a**) $P_{\mathrm{out}}$, (**b**) gain, (**c**) PAE, and (**d**) IMD. Colors are used to regroup different clouds.

Figure 5 reports the measured distributions of the main performance metrics considered in the synthesis and optimization procedure, namely $P_{\mathrm{out}}$, gain, PAE, and IMD.

It should be emphasized that the linearity metric IMD, evaluated as the ratio between the carrier power ($P_{OUT}$) and the power of the third-order intermodulation product ($P_{IM3}$), adopted in this work, is consistent with the two-tone characterization. Metrics such as adjacent channel power ratio (ACPR) and error vector magnitude (EVM) are instead specific to digitally modulated signals and depend on the selected waveform, bandwidth, modulation format, and measurement conditions. Therefore, they cannot be rigorously reported from the present pulsed/CW two-tone dataset. Thus, the calculated IMD values indicate the linearity trends with respect to the LM-ET control parameters.

As shown in Figure 5a–d, the measurement campaign provides a dense experimental mapping of the DUT response over the considered operating space, capturing the dependence of the amplifier behavior on both the control settings and the input excitation level.

Specifically, Figure 5a,b identifies the achievable output-power and gain regions, while Figure 5c,d illustrates the corresponding efficiency–linearity. From the data, we observe that the prototype exhibits a peak output power exceeding 39 dBm and a corresponding PAE exceeding 54%; the maximum IMD is about 38 dB, with a power gain exceeding 21 dB. It is worth noting that negative PAE values may occur when the power gain tends to vanish, owing to counter-phase current recombination at the summing node. This dataset, therefore, serves as the experimental basis for extracting shaping laws and identifying Pareto-optimal operating trajectories.

In the proposed LM-ET PA architecture, the operating condition is determined by a set of control parameters that enable exploration of the broad performance space typical of dual-input, supply-modulated power amplifiers. As illustrated in Figure 3, these control variables include the phase shift and input attenuation applied to the auxiliary path, together with the drain supply voltages of the main and auxiliary amplifiers. The RF input power delivered by the signal generator is swept over a predefined range and is adopted as the reference independent variable, thereby allowing the architecture to be evaluated under different excitation conditions and enabling a consistent comparison among the corresponding operating points. The actual input powers at the main and auxiliary branches are monitored using a directional coupler and a calibrated power meter. These quantities are denoted as $P_{in,M}$ and $P_{in,A}$, respectively, although only $P_{in,M}$ is used as an independent variable in the present analysis. From Figure 5, a certain spread in the measured input power can be observed across the data clouds. This effect is mainly attributed to variations in the input reflection coefficients of the prototype, which, combined with the limited directivity of the directional couplers, result in slight measurement inaccuracies.

## 4. Pareto-Driven Synthesis and Interpretation of Shaping Functions

All measured operating points were exported and post-processed in a commercial computing platform, where a dedicated analysis framework was developed to organize the complete experimental dataset and to enable the synthesis of admissible control trajectories. The dataset includes measured combinations of drain supply voltage, auxiliary-path attenuation, and auxiliary-path phase shift, thereby providing a multidimensional description of the accessible operating space of the proposed architecture.

Starting from this measured cloud, the objective is not the identification of isolated favorable operating points, but the synthesis of complete *shaping functions* governing the transmitter over the full input-power excursion. This aspect is central to the proposed methodology. In the considered dual-input LM-ET architecture, the control action is inherently dynamic and must evolve with the excitation level. Accordingly, the design problem must be formulated in functional form rather than in terms of static bias optimization.

More specifically, the control strategy is described by the triplet

(6) $$u\left(P_{\mathrm{in},M}\right)={\left[V_{D}\left(P_{\mathrm{in},M}\right),\;ATT\left(P_{\mathrm{in},M}\right),\;PH\left(P_{\mathrm{in},M}\right)\right]}^{T},$$

where $V_{D}$ denotes the drain supply voltage, while ATT and PH represent the attenuation and phase shift applied to the auxiliary path. Therefore, each candidate solution is not a single point selected from the measured cloud, but a complete triplet of shaping functions defined over the main input-power axis $P_{\mathrm{in},M}$.

This distinction has major methodological implications. A pointwise optimization of the measured multidimensional dataset would generally return, at each input power level, the locally best control setting according to the selected objective. However, such a procedure would not guarantee that the resulting sequence of operating points defines a physically realizable control law. Adjacent operating states could correspond to abrupt or mutually inconsistent changes of supply voltage, attenuation, or phase, thereby producing a trajectory that is numerically assembled *a posteriori* but physically implausible. By contrast, the purpose of the present work is to identify shaping functions that remain smooth, admissible, and meaningful over the whole dynamic range of operation.

This requirement is especially stringent for the supply trajectory $V_{D}\left(P_{\mathrm{in},M}\right)$, which is intended to represent the envelope-tracking action of the transmitter. In physical terms, the drain supply should follow the signal envelope, or equivalently, its magnitude, which is naturally associated with increasing input drive and is therefore monotonically related to $P_{\mathrm{in},M}$. At the same time, the practical feasibility of the supply law does not depend only on its amplitude excursion, but also on how rapidly it must vary during normal operation. Indeed, the rate of variation of the envelope is directly related to the instantaneous signal bandwidth, namely, to how fast the envelope changes from one sample to the next. Therefore, an admissible shaping law must not only provide suitable bias values versus $P_{\mathrm{in},M}$, but must also remain compatible with the finite dynamic capability of a realistic supply modulator.

Once mapped back to the actual signal operation, excessively abrupt variations in $V_{D}\left(P_{\mathrm{in},M}\right)$ would imply a supply modulation speed incompatible with the envelope path’s finite bandwidth.

The same applies to the auxiliary-path controls $ATT(P_{\mathrm{in},M})$ and $PH(P_{\mathrm{in},M})$, which must jointly trace a feasible and sufficiently regular path within the measured control space. Hence, the problem is intrinsically more demanding than a conventional operating-point selection problem: the unknowns are functions, and each admissible solution must preserve physical consistency along the full input-power axis while remaining compatible with the dynamic constraints of actual signal operation.

Once a shaping-function triplet is assigned, the experimental dataset is used to reconstruct the corresponding performance trajectories,

(7) $$y\left(P_{\mathrm{in},M}\right)={\left[G\left(P_{\mathrm{in},M}\right),\;P_{\mathrm{out}}\left(P_{\mathrm{in},M}\right),\;\mathrm{PAE}\left(P_{\mathrm{in},M}\right),\;\mathrm{IMD}\left(P_{\mathrm{in},M}\right),\;\mathrm{OIP}3\left(P_{\mathrm{in},M}\right)\right]}^{T},$$

where $\mathrm{OIP}3=3P_{\mathrm{out}}/2-P_{\mathrm{IM}3}/2$, with all quantities expressed in dBm, so that each candidate shaping solution is mapped into a corresponding performance trajectory. As a consequence, each point appearing in the objective space must be interpreted as the image of one complete shaping-function triplet, namely one full transmitter control strategy, and not as the representation of a single static operating condition.

The optimization is performed on the experimentally characterized LM-ET dataset. For each input-envelope level, the drain supply voltage and the auxiliary RF amplitude/phase pair are selected by evaluating the available operating points in terms of output power, efficiency, and linearity constraints. This procedure transforms the measured static characterization into envelope-dependent control functions, which can be applied sample-by-sample or envelope-bin-by-envelope-bin, to a modulated waveform.

### 4.1. Statistical Evaluation Under a Rayleigh Envelope Model

A second key feature of the proposed framework is the statistical evaluation of the relevant figures of merit, consistently with modulated-signal operation. The signal envelope is therefore modeled as Rayleigh distributed, as expected for a complex Gaussian baseband waveform.

Denoting by *r* the normalized signal-envelope amplitude, the corresponding probability density function is

(8) $$f_{r}\left(r\right)=\frac{r}{\sigma^{2}}\exp\left(-\frac{r^{2}}{2\sigma^{2}}\right),\;r\geq 0,$$

where the scale factor is determined by the selected peak-to-average power ratio (PAPR), which relates the maximum meaningful envelope excursion to the average operating condition and defines the weighting law for each admissible shaping trajectory.

After discretization over the sampled $P_{\mathrm{in},M}$ axis, the continuous density in (8) is converted into a set of normalized weights $\left\{w_{k}\right\}$ satisfying

(9) $$\sum_{k=1}^{K_{\max}}w_{k}=1,$$

where $K_{\max}$ denotes the highest meaningful input-power sample of the considered trajectory. For any quantity $\Psi_{k}=\Psi \left(P_{\mathrm{in},M,k}\right)$, the PDF-aware mean value is

(10) $$\bar{\Psi }=\sum_{k=1}^{K_{\max}}w_{k}\;\Psi_{k}.$$

Thus, output power, efficiency, and linearity are optimized through statistically weighted averages, such as ${\bar{P}}_{\mathrm{out}}$, $\bar{\mathrm{PAE}}$, and $\bar{\mathrm{IMD}}$, rather than through isolated drive-level values. This prevents low-probability operating regions from driving the synthesis.

### 4.2. Flat-Gain Objective and Detection of the Linear Region

Gain is treated differently because its desired behavior differs from that of the other metrics. In a power amplifier, gain should remain approximately constant up to the onset of compression. Therefore, the gain-related objective is not averaged over the full input-power range, but is associated with the flat-gain region, defined as the largest initial interval where the gain remains compatible with the prescribed quasi-constant behavior.

Let $G_{k}$ denote the gain samples reconstructed along a candidate shaping trajectory. The flat-gain region is identified as

(11) $$\Omega_{\mathrm{flat}}=\left\{1,\ldots ,K_{\mathrm{flat}}\right\},$$

where $K_{\mathrm{flat}}$ is the largest index of the initial input-power interval over which the gain remains within the prescribed tolerance around the target value. Once this interval has been identified, a representative flat-gain metric is extracted as

(12) $$G_{\mathrm{flat}}=\frac{1}{K_{\mathrm{flat}}}\sum_{k=1}^{K_{\mathrm{flat}}}G_{k},$$

which provides a compact descriptor of the amplifier behavior in its linear operating region.

This definition adds a constraint to the synthesis: candidate shaping functions must improve efficiency, output power, and linearity in a statistically meaningful sense while preserving a sufficiently extended and regular flat-gain region. The optimization is therefore driven by heterogeneous metrics with distinct physical meanings, rather than by a single scalar objective.

### 4.3. Strongly Competing Objectives and Meaning of the Pareto Fronts

Under the above premises, the synthesis problem is inherently multi-objective and strongly conflicting. The maximization of ${\bar{P}}_{\mathrm{out}}$, $\bar{\mathrm{PAE}}$, $\bar{\mathrm{IMD}}$, and $G_{\mathrm{flat}}$ leads to requirements that cannot, in general, be improved simultaneously.

This competition is rooted in the physics of the underlying amplifier. Increasing the average output power typically pushes the device toward more aggressive operating conditions, which may reduce the extent of the flat-gain region and worsen linearity. Improving average efficiency may require supply and load conditions that are advantageous from an energy standpoint, but detrimental to gaining flatness or spectral behavior. Conversely, enforcing better linearity usually requires more conservative control trajectories, with possible penalties in both output power and efficiency. Therefore, the objectives are not merely distinct numerical targets, but structurally competing requirements that generate an antagonistic multi-objective landscape.

For this reason, the correct output of the synthesis is not a single optimum, but a set of non-dominated shaping solutions. In the present framework, a shaping-function triplet is Pareto-optimal if no other admissible triplet can improve at least one objective without degrading at least one of the others. The resulting Pareto fronts, therefore, represent the locus of the best physically achievable tradeoffs offered by the considered architecture.

This interpretation is crucial. The Pareto fronts are not an accessory graphical summary of the optimization process, but the actual design-oriented output of the methodology. Each point on a front corresponds to one complete and physically realizable shaping strategy, that is, one full triplet $u(P_{\mathrm{in},M})$. Moving along a Pareto front does not simply mean changing the numerical values of a few scalar metrics; it means replacing one admissible family of control laws with another, thereby moving from one global transmitter behavior to another.

Accordingly, the Pareto fronts provide a direct map of the achievable compromises among the considered objectives. Extreme points identify shaping strategies that deliberately privilege one design goal, such as efficiency enhancement, output-power capability, or gain regularity, at the expense of the others. Intermediate points, and especially knee regions, identify more balanced solutions, where a limited degradation of one figure of merit allows a comparatively large improvement of another. In this sense, the fronts do not merely state that tradeoffs exist; they quantify their severity and reveal where the most convenient design compromises are located.

Equally important, the fronts compactly represent the global design freedom offered by the proposed LM-ET architecture. Broad and well-populated fronts indicate that the architecture admits a wide family of distinct yet admissible control strategies. Steep or compressed regions, instead, indicate that the corresponding objectives are strongly locked by the device physics, so that even substantial modifications of the shaping laws produce only limited improvement. The Pareto representation is therefore informative not only about which solutions are non-dominated, but also about how flexible the architecture is with respect to different design priorities.

**Figure 6:**
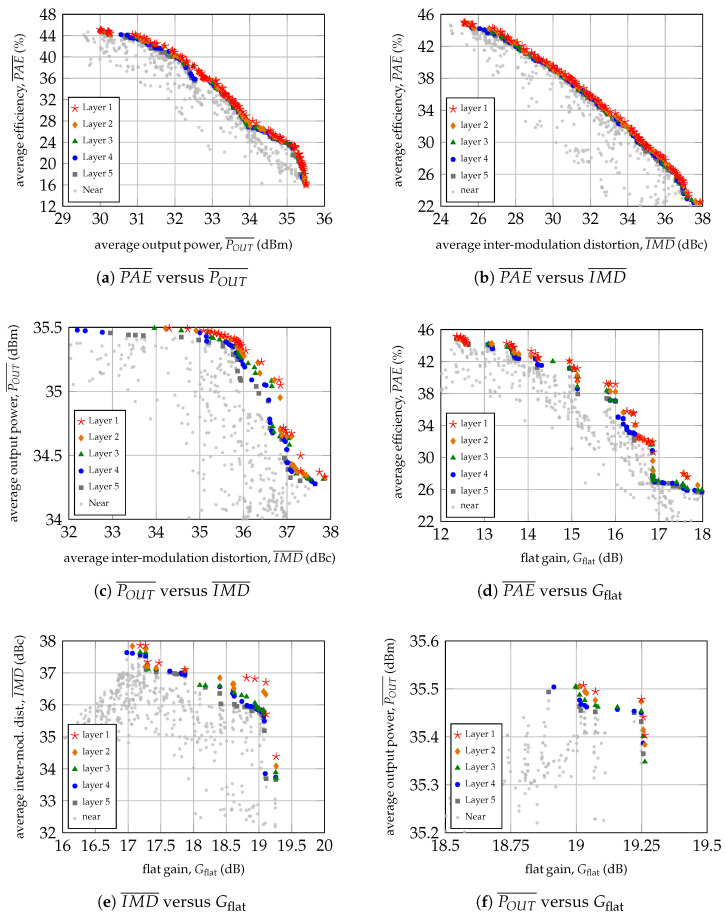
Representative Pareto diagrams obtained from the customized multi-objective synthesis of admissible shaping-function triplets. Each point corresponds to one complete control strategy $u(P_{in,M})$. The highlighted fronts identify the non-dominated shaping solutions and directly quantify the achievable tradeoffs among $G_{\mathrm{flat}}$, $\bar{P_{OUT}}$, $\bar{PAE}$, and $\bar{IMD}$.

The pairwise diagrams reported in Figure 6 should be interpreted precisely in this way. The $\bar{\mathrm{PAE}}$–${\bar{P}}_{\mathrm{out}}$ plane highlights the energetic cost associated with pushing the transmitter toward higher average delivered power. The $\bar{\mathrm{PAE}}$–$\bar{\mathrm{IMD}}$ plane reveals the efficiency-linearity competition and shows how aggressively the control laws can be shaped before the linearity-related metric is significantly affected. The $G_{\mathrm{flat}}$–${\bar{P}}_{\mathrm{out}}$ plane quantifies the compromise between maintaining a broad flat-gain region and extracting larger average output power. Finally, the $G_{\mathrm{flat}}$–$\bar{\mathrm{IMD}}$ plane makes explicit the relation between gain regularity and spectral behavior. Taken together, these fronts provide a structured interpretation of the admissible shaping laws and constitute the natural basis for selecting representative solutions for deeper inspection.

This point also clarifies the role of the following section. The following Section 5 does not establish the existence of the tradeoffs, which are already encoded and made explicit by the Pareto fronts. Rather, the subsequent analysis examines a limited number of representative shaping trajectories selected from different front regions to show how the corresponding control functions and performance evolutions concretely realize the tradeoffs predicted by the Pareto-based synthesis.

### 4.4. Customized Multi-Objective Invasive Weed Optimization

Because of the functional nature of the design variables and the strong competition among the objectives, the synthesis problem is highly non-convex and cannot be effectively addressed through simple pointwise search or through a naive scalarization of the objectives. The search space is broad, irregular, and constrained by physical admissibility. Moreover, each candidate must be evaluated as a complete control trajectory reconstructed from measured data, rather than as an isolated operating point. This makes the optimization stage particularly demanding.

To tackle this problem, a customized multi-objective Invasive Weed Optimization (IWO) strategy was adopted [20,21,22,23,24]. IWO-based methods are well-suited to non-convex electrical and electromagnetic design problems with competing objectives  [25,26]. This makes them suitable for the present framework, where each candidate is a complete shaping-function triplet, and its quality is evaluated only after trajectory reconstruction and multi-objective assessment  [26,27].

IWO has also been applied to PAPR reduction in communication signals  [28,29]. This is relevant here because PAPR defines the signal statistics used to weight the shaping functions, as discussed in Section 4.1.

The main numerical quantities involved in the customized IWO synthesis are summarized in Table 2. The first group of parameters describes the experimental operating map, while the second group reports the IWO hyperparameters used to control the population, the number of generations, the reproduction process, the dispersal law, and the archive-stability criterion. The table also reports the maximum number of objective-function evaluations allowed by the final massive IWO setting; the actual number of evaluations is logged for each optimization case and can be lower when the stall criterion is reached.

The population is initialized with $N_{\mathrm{pop},0}$ admissible shaping triplets and is allowed to grow up to $N_{\mathrm{pop},\max}$ individuals before competitive exclusion. The evolution is run for at most $N_{\mathrm{it}}$ generations and is repeated over $N_{\mathrm{run}}$ independent runs. Each individual generates a number of seeds between $S_{\min}$ and $S_{\max}$, depending on its Pareto rank and diversity contribution. The dispersal spread decreases from $\sigma_{\mathrm{ini}}$ to $\sigma_{\mathrm{fin}}$ according to the exponent $\eta_{\sigma }$, so that early generations favor exploration and late generations favor local refinement.

Each IWO individual represents one complete shaping-law triplet rather than an isolated operating point. The three control laws $V_{D}\left(P_{\mathrm{in},M}\right)$, $ATT(P_{\mathrm{in},M})$, and $PH(P_{\mathrm{in},M})$ are encoded by normalized control knots and then interpolated over the measured input-power grid. The drain-voltage law is constrained to be monotonically non-decreasing with $P_{\mathrm{in},M}$, and its maximum variation between adjacent input-power samples is limited to avoid unphysical supply jumps. In the final implementation, the maximum allowed step of the reconstructed $V_{D}\left(P_{\mathrm{in},M}\right)$ law is 1 V between adjacent input-power samples. This constraint enforces the envelope-tracking interpretation of the supply trajectory and prevents the optimizer from exploiting discontinuous laws that would not be compatible with a realistic envelope modulator.

The flat-gain interval used inside the IWO is detected with the same logic as in the reference deterministic synthesis. Starting from the lowest input-power sample, the interval is extended as long as the reconstructed gain remains within the prescribed tolerance around the target gain. No additional peak-to-peak ripple constraint is imposed.

Convergence is assessed at the level of the non-dominated archive. The optimization stops when either $N_{\mathrm{it}}$ is reached, or the Pareto archive shows no appreciable variation over the last $N_{\mathrm{stall}}$ generations, according to the tolerance $\varepsilon_{\mathrm{stall}}$. The computational cost is mainly due to trajectory reconstruction. In the present dataset, each measured quantity is stored on a 33×31×22×15 operating map, corresponding to drain supply, input power, auxiliary attenuation, and auxiliary phase, respectively. Each candidate shaping triplet therefore defines a trajectory with at most $K_{\max}\leq 31$ input-power samples. If $N_{\mathrm{eval}}$ candidate shaping triplets are evaluated, the dominant cost scales approximately as $O(N_{\mathrm{eval}}K_{\max})$, in addition to dominance sorting, competitive exclusion, and archive update. With the settings of Table 2, the theoretical upper bound is

$$N_{\mathrm{eval},\max}=N_{\mathrm{run}}\left(N_{\mathrm{pop},0}+N_{\mathrm{it}}\;N_{\mathrm{pop},\max}\;S_{\max}\right)=1,920,400$$

evaluated shaping laws per optimization case. The actual number of evaluations, elapsed runtime, runtime per evaluation, final archive size, and stopping reason are logged for each case and used to quantify the computational burden of the synthesis.

The sensitivity to the main hyperparameters is assessed by comparing nominal and massive exploration settings. The parameters with the largest impact are the maximum population size, the maximum number of generations, the archive size, and the initial/final dispersal range. Increasing $N_{\mathrm{pop},\max}$, $N_{\mathrm{it}}$, and $N_{\mathrm{arch}}$ mainly increases the density of the non-dominated archive and improves the sampling of extreme Pareto regions, especially in the high-gain and high-linearity portions of the objective space. This improvement is obtained at the cost of an almost proportional increase in the number of evaluated shaping laws. The dispersal parameters control the exploration/refinement balance: excessively small $\sigma_{\mathrm{ini}}$ values favor premature convergence toward local families of shaping laws, whereas excessively large values delay the stabilization of flat-gain-feasible trajectories. The final massive setting is therefore selected as a compromise between Pareto-front density, repeatability of the selected representative regions, and computational cost.

In the proposed framework, each individual of the population encodes one complete shaping-function triplet as in (6). The optimizer, therefore, acts directly in the space of admissible control laws. For each candidate, the measured cloud is interrogated to reconstruct the corresponding trajectories of gain, output power, efficiency, intermodulation distortion, and OIP3, as in (7). The flat-gain region is then automatically detected, the PDF-aware mean metrics are computed, and the candidate is finally associated with the objective vector

(13) $$J={\left[G_{\mathrm{flat}},\;{\bar{P}}_{\mathrm{out}},\;\bar{\mathrm{PAE}},\;\bar{\Delta }\right]}^{T},\;\Delta =P_{\mathrm{out}}-P_{\mathrm{out},3}.$$

All four components of J are maximized. The overbar denotes the Rayleigh-PDF-weighted average over the reconstructed input-power trajectory, whereas $G_{\mathrm{flat}}$ is evaluated only over the detected flat-gain interval. The quantity $\bar{\Delta }$ is the linearity-related metric used in the implementation and is reported in the following Pareto plots and tables as the average IMD indicator.

The customization of the IWO framework lies in this problem-oriented formulation: individuals are complete shaping laws, fitness is evaluated from reconstructed trajectories and statistically weighted metrics, and the output is a non-dominated set from which the Pareto fronts are extracted.

Thus, the customized IWO is used as a synthesis engine for Pareto fronts of control functions. Its role is not merely to search for numerically favorable candidates, but to populate the feasible objective space with physically admissible shaping strategies, so that the Pareto structure of the problem can emerge clearly.

A high-level representation of the adopted procedure is summarized in Algorithm 1. The emphasis is intentionally placed on the methodology’s logical structure, since the main contribution lies in formulating a Pareto-driven synthesis problem in which the unknowns are shaping functions and the objective landscape is reconstructed from measured multidimensional data.

The proposed methodology therefore produces, for each Pareto-optimal solution, both the control trajectories $\left(V_{D},ATT,PH\right)$ and the corresponding performance trajectories $\left(G,P_{\mathrm{out}},\mathrm{PAE},\Delta ,\mathrm{OIP}3\right)$ as functions of $P_{\mathrm{in},M}$. The same linearity-related quantity $\Delta =P_{\mathrm{out}}-P_{\mathrm{out},3}$ is consistently used for the IMD-oriented objective and for the corresponding Pareto interpretation. This representation directly links each point on the Pareto front to one complete and physically realizable shaping strategy, thereby turning the Pareto fronts themselves into the primary interpretation tool of the synthesis.
**Algorithm 1** Customized multi-objective synthesis of Pareto-optimal shaping functions  1:**Input:** measured multidimensional dataset, target gain, flat-gain tolerance, PAPR, control constraints  2:**Input:** IWO parameters $\{N_{\mathrm{pop},0},N_{\mathrm{pop},\max},N_{\mathrm{it}},N_{\mathrm{run}},S_{\min},S_{\max},\sigma_{\mathrm{ini}},\sigma_{\mathrm{fin}},\eta_{\sigma },N_{\mathrm{stall}},\varepsilon_{\mathrm{stall}},$$N_{\mathrm{arch}}\}$  3:Define the shaping-function triplet $u\left(P_{\mathrm{in},M}\right)={\left[V_{D},ATT,PH\right]}^{T}$  4:Encode the triplet by normalized control knots and enforce the admissibility of $V_{D}\left(P_{\mathrm{in},M}\right)$, including monotonicity and maximum-step limitation  5:Build the Rayleigh-based PDF associated with the selected PAPR  6:Map the PDF onto the discrete $P_{\mathrm{in},M}$ support and compute the normalized weights $\left\{w_{k}\right\}$  7:Initialize a customized multi-objective IWO population of admissible shaping-function triplets  8:**for** each candidate shaping triplet **do**  9:   Reconstruct *G*, $P_{\mathrm{out}}$, PAE, $\Delta =P_{\mathrm{out}}-P_{\mathrm{out},3}$, and OIP3 from the measured dataset10:   Detect the flat-gain region and compute $G_{\mathrm{flat}}$11:   Compute ${\bar{P}}_{\mathrm{out}}$, $\bar{\mathrm{PAE}}$, and $\bar{\Delta }$ using the PDF weights12:   Associate the candidate shaping triplet with the objective vector J13:**end for**14:Initialize the non-dominated archive15:**for** each generation up to $N_{\mathrm{it}}$ **do**16:   Rank the population according to Pareto dominance and diversity17:   Assign a number of seeds between $S_{\min}$ and $S_{\max}$ to each individual18:   Generate offspring by dispersing the parent shaping laws with the current spread σ19:   Enforce control bounds and admissibility constraints on the offspring20:   Evaluate offspring trajectories and objective vectors21:   Merge parents and offspring, update the non-dominated archive, and limit it to $N_{\mathrm{arch}}$ solutions22:   Apply competitive exclusion to limit the population to $N_{\mathrm{pop},\max}$ individuals23:   Check archive stabilization over $N_{\mathrm{stall}}$ generations24:**end for**25:Collect the non-dominated shaping functions26:Reconstruct and identify the Pareto fronts in the objective space27:Select representative shaping functions for subsequent trajectory analysis

## 5. Analysis of Representative Pareto-Optimal Shaping Strategies

Once the Pareto fronts have been extracted, the proposed optimization framework provides a structured set of admissible shaping solutions, each corresponding to a complete transmitter control strategy. The purpose of this section is not to further prove the existence of trade-offs, which is already made explicit by the Pareto-based synthesis, but rather to clarify how these trade-offs are physically realized by representative solutions. In this sense, the following discussion translates the geometric structure of the Pareto fronts into the corresponding behavior of the LM-ET architecture.

To this end, a set of Pareto-representative cases has been selected from different regions of the non-dominated fronts. These solutions span different design priorities, ranging from output-power- and gain-oriented strategies to more balanced compromises that balance efficiency and linearity. By inspecting these representative points, it becomes possible to understand how different locations on the Pareto fronts correspond to different shaping laws, and how these shaping laws, in turn, determine different evolutions of the main performance figures along the input-power axis.

**Figure 7:**
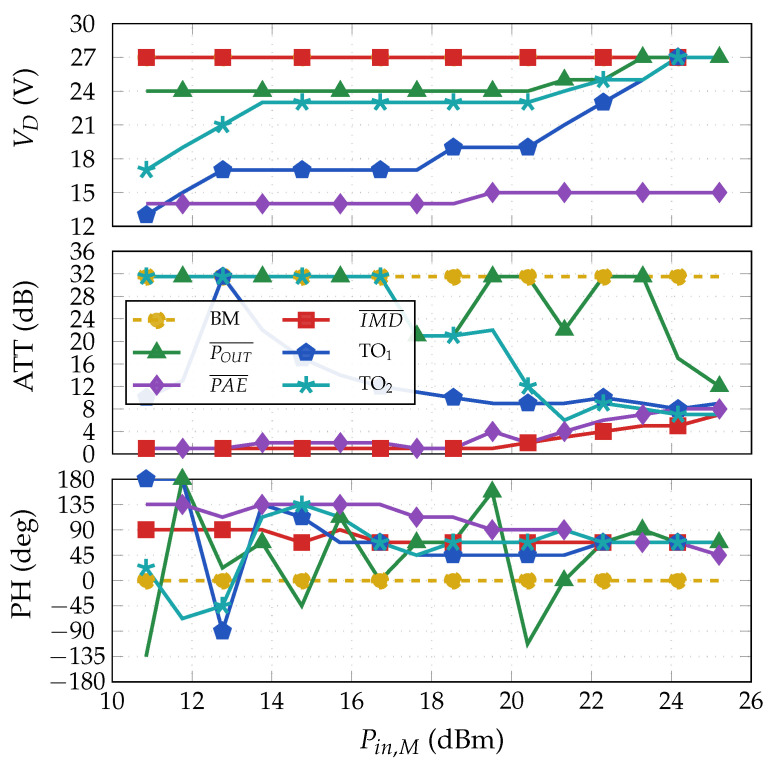
Representative shaping-function triplets associated with selected Pareto-optimal solutions. The reported trajectories describe the evolution of the control variables as functions of $P_{\mathrm{in},M}$ and provide the physical implementation of the different trade-offs identified on the Pareto fronts.

Figure 7 reports the shaping functions associated with the selected Pareto-representative cases. These curves provide a direct view of the control strategies synthesized by the proposed framework, namely the coordinated evolution of drain supply voltage, auxiliary-path attenuation, and auxiliary-path phase shift as functions of the main input power. Since each Pareto point corresponds to one complete shaping-function triplet, the curves in Figure 7 should be interpreted as the actual control laws implementing the trade-offs identified in the objective space. Their comparison is especially informative because it reveals how different optimality criteria yield distinct families of admissible control trajectories.

**Figure 8:**
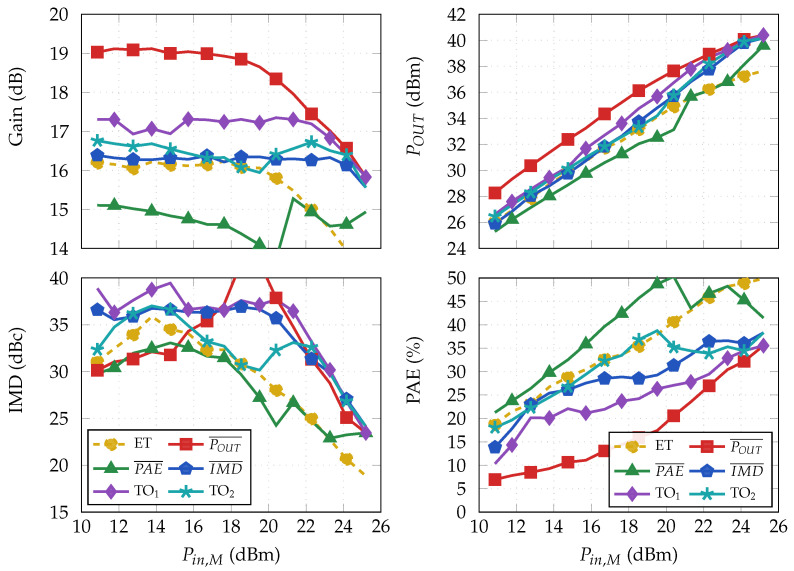
Resulting performance trajectories obtained by applying the shaping functions reported in Figure 7. The curves highlight how different Pareto-optimal control laws translate into distinct compromises among gain behavior, output power capability, efficiency, and linearity.

Figure 8 shows the corresponding performance trajectories obtained when the shaping functions of Figure 7 are applied to the measured dataset. In this way, the effect of each synthesized control strategy can be directly evaluated in terms of gain, output power, efficiency, and linearity over the entire input-power excursion. The combined reading of Figure 7 and Figure 8 is particularly useful: the former describes the control action, whereas the latter shows the resulting amplifier behavior. Together, they provide a physically transparent interpretation of the Pareto-optimal solutions and clarify how changes in the shaping laws are reflected in the corresponding performance trajectories.

Different from the static/two-tone validation reported in [16], the present validation evaluates the behavior of the optimized driving laws along the envelope trajectory of a realistic modulated signal. The LM-ET mechanism is therefore not tuned at a single back-off condition, but is controlled as a function of the instantaneous signal envelope. The reported average output power, efficiency, and linearity-related figures are obtained by applying the synthesized shaping functions to the measured LM-ET operating map.

A further, particularly relevant part of the discussion is the comparison with a conventional envelope-tracking benchmark. This reference provides a meaningful baseline for assessing the actual benefit of the proposed LM-ET strategy relative to a more conventional supply-modulated operation. The purpose of the comparison is not to identify a universally superior operating point in absolute terms, but rather to quantify how the additional degrees of freedom introduced by the dual-input load-modulated architecture reshape the attainable trade-off space.

Table 3 summarizes this comparison by collecting the selected LM-ET special cases together with the benchmark reference. More than a simple numerical summary, the table offers a compact view of the shaping versatility enabled by the proposed framework. An immediate result is that a large portion of the LM-ET solutions simultaneously improve gain-related indicators, mean and maximum output power, and linearity-related quantities relative to the benchmark. For instance, *BestMeanPout* increases the mean and maximum output power from 33.0/37.7 to 35.5/40.5 dBm, while also improving mean IMD from 31.0 to 34.0 dB and mean OIP3 from 18.5 to 22.5 dBm. Similarly, *BestGain* reduces the gain-error indicator from 3.88 to 3.74 while still achieving 35.3 dBm mean output power, and 22.2 dBm mean OIP3. These results confirm that the additional shaping freedom offered by the proposed architecture can shift the operating trajectory into regions inaccessible to the benchmark when output power, gain regularity, and spectral behavior are prioritized.

At the same time, the table shows that these advantages are not free. In most reported LM-ET cases, improvements in gain behavior, power capability, and linearity are accompanied by a reduction in PAE relative to the benchmark. This is fully consistent with the Pareto interpretation discussed in the previous section: the objectives are genuinely competing, and improving one group of metrics generally requires accepting a penalty in another. The clearest counterexample is *BestMeanPAE*, which is the only case that distinctly improves both mean and maximum PAE, from 0.33/0.50 to 0.45/0.53, but does so at the expense of output power, IMD, and OIP3. This result is important because it shows that efficiency enhancement is not excluded by the proposed LM-ET architecture, but can be explicitly recovered whenever it is selected as the dominant design priority.

The pairwise Pareto cases are especially useful because they make the trade-offs directly readable along a single front. In particular, the 25/75, 50/50, and 75/25 solutions show how the operating point moves in a controlled manner when the relative priority between two competing objectives is progressively changed. This is clearly visible on the *Pout–PAE* front. Moving from *MidFront_25Pout_75PAE* to *MidFront_50Pout_50PAE* and then to *MidFront_75Pout_25PAE*, the mean output power increases from 31.9 to 33.3 to 34.6 dBm, whereas mean PAE decreases from 0.41 to 0.33 to 0.25. Thus, the 50/50 point is not simply an arithmetic midpoint: it is the interior Pareto solution that preserves a substantial fraction of the power improvement without incurring the full efficiency penalty associated with the more power-oriented end of the front.

A similar behavior emerges on the *PAE–IMD* front. The transition from *MidFront_75PAE_25IMD* to *MidFront_50PAE_50IMD* and then to *MidFront_25PAE_75IMD* moves mean PAE from 0.41 to 0.35 to 0.29, while mean IMD increases from 29.1 to 32.1 to 35.2 dB. Here again, the 50/50 case identifies the interior part of the Pareto front where both metrics remain reasonably preserved, whereas the 25/75 and 75/25 points reveal the two opposite directions of the trade-off. The same interpretation applies to the *Pout–IMD* and *PAE–Gain* fronts: the 25/75 and 75/25 solutions expose the two competing tendencies, while the 50/50 solution marks the region in which the trade-off is most directly usable when neither objective can be treated as secondary.

From a multi-objective viewpoint, these pairwise interior points are important because they show that the proposed synthesis does not merely recover isolated extremes, but actually resolves the geometry of the Pareto fronts. In other words, the framework not only tells the designer which endpoint maximizes one objective or the other, but also provides intermediate non-dominated solutions that quantify how much one metric must be traded off to recover another. This is precisely the type of information that makes Pareto synthesis practically valuable.

Particularly significant, however, are the two 4D compromise solutions, *BestBalanced4D* and *BestKnee4D*, because, as shown in Table 3, they identify two different and highly informative interior regions of the full four-objective Pareto set.

*BestBalanced4D* is especially relevant because it is the solution where the improvement is distributed across several objectives at once, without requiring any severe collapse of the others. Relative to the benchmark, the gain-flatness metric is essentially preserved and even slightly improved, from 17.0 to 17.3, while mean and maximum output power increase from 33.0/37.7 to 34.2/40.4 dBm. At the same time, mean and maximum IMD improve from 31.0/38.7 to 36.4/39.4 dB, and mean and maximum OIP3 rise markedly from 18.5/19.0 to 22.4/26.0 dBm. The price paid is a reduction in mean and maximum PAE from 0.33/0.50 to 0.25/0.36, but this penalty remains clearly less severe than in strongly power-oriented solutions such as *BestMeanPout*, where mean PAE falls to 0.16. The specific interest of *BestBalanced4D* therefore lies in its ability to deliver simultaneous improvements in output power, linearity, and OIP3, while keeping gain flatness substantially unchanged and avoiding an excessively severe efficiency penalty.

*BestKnee4D* has a different meaning. It identifies the region of the 4D Pareto set where the exchange among objectives is still favorable, but is about to become markedly more expensive. Relative to the benchmark, it preserves the gain-flatness metric with only a limited reduction from 17.0 to 16.7, a gain in mean and maximum output power from 33.0/37.7 to 33.5/40.2 dBm, and a clear improvement in mean and maximum OIP3 from 18.5/19.0 to 19.9/24.5 dBm. Its efficiency remains much closer to the benchmark than in *BestBalanced4D*, with mean and maximum PAE equal to 0.32/0.39 versus 0.33/0.50 for the benchmark, while the linearity improvement is more selective: mean IMD increases from 31.0 to 32.8 dB, whereas maximum IMD slightly decreases from 38.7 to 37.0 dB. This is precisely why the knee is meaningful in Pareto theory: it marks the last region where one can still obtain tangible gains in power and OIP3 while paying only a limited price in the remaining metrics. Beyond this point, further improvements are possible only at the cost of much greater degradation in efficiency or other objectives. In this sense, *BestKnee4D* marks the onset of diminishing returns on the non-dominated set.

Taken together, the pairwise 25/75–50/50–75/25 solutions and the two 4D interior solutions show that the proposed framework does not simply identify a few extreme optima. Rather, it reconstructs a family of physically meaningful operating conditions distributed across the trade-off space: endpoints that reveal the attainable limits, pairwise interior points that quantify the exchange between two objectives, balanced 4D points that prevent any metric from becoming unacceptable, and knee points that indicate where the marginal cost of further improvement becomes too high. This is the main practical strength of the proposed LM-ET approach.

Overall, the results discussed in this section support the effectiveness of the proposed method in two complementary senses. First, they show that the Pareto-based framework generates physically meaningful shaping laws, rather than merely abstract optimal points in objective space. Second, they demonstrate that the additional control freedom offered by the LM-ET architecture can be translated into tangible performance advantages relative to a conventional envelope-tracking reference, especially when output-power capability, gain regularity, and linearity are primary design targets.

Finally, it should be noted that the present validation is based on an experimentally grounded quasi-static characterization of the LM-ET PA. Therefore, it captures the envelope-dependent trade-off among output power, efficiency and linearity, but it does not fully include dynamic effects associated with the finite bandwidth of the envelope modulator, the auxiliary-path control bandwidth, synchronization errors, or long-term memory effects. These aspects will be addressed in a future fully dynamic wideband implementation.

## 6. Discussion and Conclusions

This work has shown that he proposed dual-input envelope-tracking and load-modulated architecture is not merely an alternative implementation of a conventional ET PA, but a substantially more flexible transmitter solution capable of realizing operating regimes inaccessible to the benchmark case. The main outcome is therefore not the identification of a single optimal operating point, but the demonstration that the proposed LM-ET approach opens a broad, highly structured space of physically realizable trade-offs among gain flatness, output power capability, efficiency, and linearity.

This result emerges very clearly from Table 3. Compared with the benchmark, the proposed framework can move the transmitter toward markedly different operating regions depending on the selected design priority. For instance, power-oriented solutions such as *BestMeanPout* and *MidFront_Pout_Gain* increase the mean output power from 33.0 to 35.5 dBm and the maximum output power from 37.7 to 40.5 dBm, while also raising the flat-gain metric from 17.0 to about 19.0 dB. At the same time, the flat operating range can be significantly extended: $P_{in,M,max-flat}$ grows from 11.8 up to 24.2 in the *MidFront_Pout_PAE* case, which is a remarkable enlargement of the usable quasi-linear region. Likewise, the gain error can be drastically reduced, with Max$|e_{G}|$ decreasing from 3.88 in the benchmark down to 0.37 in the same case, thus confirming that the proposed shaping strategy can enforce very regular gain behavior over a much wider operating interval.

The table also shows that the proposed architecture enables particularly interesting compromises that go well beyond a simple “power versus efficiency” trade-off. Several solutions simultaneously improve output power, gain-related figures, and linearity-related figures relative to the benchmark. For example, *BestMeanIMD* raises the mean IMD metric from 31.0 to 37.9 and the mean OIP3 from 18.5 to 23.3 dB, while still increasing the mean output power to 34.3 dBm and reducing Max$|e_{G}|$ from 3.88 to 1.64. Similarly, *BestGain* achieves a flat-gain value of 19.3 dB, extends $P_{in,M,max-flat}$ to 15.7, and increases the maximum OIP3 from 19.0 to 28.0 dB. These are not marginal corrections; they indicate that the additional degrees of freedom introduced by the dual-input load-modulated ET architecture enable the synthesis of highly specific and highly valuable operating profiles tailored to distinct system-level priorities.

Equally important, the proposed framework is not restricted to aggressive power- or linearity-oriented solutions. When efficiency is chosen as the dominant objective, the synthesis can deliberately steer the same hardware toward a different regime. This is clearly visible in the *BestMeanPAE* case, where the mean PAE increases from 0.33 to 0.45 and the maximum PAE from 0.50 to 0.53, and also in weighted cases such as *MidFront_75PAE_25Gain*, where the mean PAE reaches 0.42. Although these solutions sacrifice some of the gains in output power and linearity, they make an equally important point: the proposed architecture does not enforce a fixed performance profile; instead, it allows the designer to intentionally select where to place the transmitter on the trade-off surface.

This is arguably the most relevant contribution of the paper. The proposed LM-ET methodology does not merely improve one metric at the expense of all others in a rigid, predetermined manner. Instead, it enables the synthesis of uncommon and practically meaningful compromises, including solutions with up to +2.5 dB mean output-power improvement, up to +3.7 dB maximum output-power improvement, up to about +2.3 dB flat-gain enhancement, up to +5.0 dB mean OIP3 improvement, and up to +9.0 dB maximum OIP3 improvement with respect to the benchmark, while also allowing efficiency-oriented operating modes whenever required. This ability to navigate the design space and extract different families of Pareto-optimal shaping laws from the same hardware platform makes the proposed solution especially attractive for realistic RF transmitters, where the preferred balance among efficiency, linearity, gain behavior, and delivered power depends on the specific signal and application scenario.

From a methodological viewpoint, the paper has also shown that the relevant design object in this class of amplifiers is not the isolated operating point, but the shaping-function triplet itself. By combining measured multidimensional data, Rayleigh-weighted statistical evaluation, and customized Pareto-driven synthesis, the proposed framework directly associates each Pareto point with a complete, physically meaningful control strategy. In this sense, the extracted Pareto fronts are not just descriptive plots, but an actionable design map of the operating modes enabled by the proposed architecture.

Overall, the results demonstrate that the proposed LM-ET solution significantly expands the achievable performance space compared to conventional ET operation. Its real strength lies in the ability to engineer highly specific and particularly interesting compromises that would be difficult, or even impossible, to obtain with standard single-input ET approaches. Once the dual-RF-input LM-ET architecture introduced in [16] is available, a further degree of freedom is provided by the optimized envelope-dependent driving of the main supply and auxiliary RF path.

Future work will extend the framework by explicitly including dynamic constraints on control paths, considering wider-band modulated signals, auxiliary-path memory effects, and by integrating linearization-aware criteria to further strengthen the practical applicability of the proposed synthesis methodology.

## Figures and Tables

**Table 1:** Comparison with representative PA architectures and characterization approaches related to supply modulation, load modulation, dual-input operation, and probability-distribution-aware PA design.

Ref.	Amplifier Category	Freq. Band	Main Technique	Other Concrete Metric (s)	Efficiency	Max./Reported $P_{\mathrm{out}}$	Linearity	Reported Contribution
[4]	Doherty PA	550–770 MHz	Passive LM	31% FBW; 8-MHz DVB-T; high-PAPR signal	>46% average efficiency	250-W peak class; 48 dBm average	n.a.	Wideband high-power LDMOS Doherty PA.
[5]	Polar PA	1.75 GHz	EER	0.18-μm CMOS; GSM- EDGE; integrated AM	34% CW PAE; 22% average PAE	27 dBm CW; 23.8 dBm average	ACPR = −56.7/−64 dBc	Integrated polar CMOS PA with on-chip AM.
[6]	Supply-modulated	2 GHz	Class-G supply modulation	130-nm CMOS; 3.3/1.65-V supplies; 64-QAM OFDM	69% peak PAE; 22.6% average PAE	29.3 dBm peak; 19.6 dBm average	2.5% rms EVM	Class-G supply modulator with class-E PA.
[7]	ET PA	950 MHz	Dynamic supply control	GaAs MESFET; 10-MHz dc–dc converter; IS-95 CDMA	Efficiency improvement factor >1.64	1 W CW	Meets IS-95 ACPR requirement	Early ET PA with dynamic supply and gate bias.
[8]	ET modeling	850 MHz	X-parameter modeling	GaAs PA; 2-port/3-port X-parameter comparison	Average DE estimated; value n.a.	40 dBm nominal peak	ACLR estimated; value n.a.	Behavioral modeling for ET PA simulation.
[9]	Dual-band ET PA	870/2140 MHz	PDF-conscious ET design	GaN PA; WCDMA 3GPP DL signal statistics	n.a.	240 W; peak levels >54 dBm class	n.a.	Dual-band ET PA using signal-probability information.
[10]	ET characterization	n.a.	PDF-conscious characterization	GaN HEMT; pulsed harmonic load-pull; PDF-aware maps	DE from PDF- weighted maps; value n.a.	$P_{\mathrm{out}}$ from PDF-weighted maps; value n.a.	n.a.	Characterization method for optimum ET PA design.
[11]	Doherty	n.a.	AB–C Doherty theory	AB main; class-C auxiliary; tuned-load harmonics	Only analytical formulation	n.a.	n.a.	Closed-form design basis for AB–C Doherty operation.
[12]	LMBA	Broadband	Active LM	Balanced-PA concept; auxiliary RF control signal	n.a.	n.a.	n.a.	Active LM through auxiliary RF amplitude/phase.
[13]	SLMBA / balanced PA	1.8–3.8 GHz	Supply and load modulation	100-MHz signal; 10-dB PAPR; 8-level supply modulator	22.4–43.9% average composite PAE	34 dBm average	∼−50 dBc ACLR after DPD	Balanced PA with supply and active load modulation.
[14]	Dual-RF-input PA	1–3 GHz	Doherty–outphasing continuum	6.7-dB PAPR WCDMA; two 15-W GaN HEMTs	45% PAE at 6-dB OBO; 40–50% PAE after DPD	44±0.9 dBm	<−57 dBc ACLR after DPD	Dual-input PA between Doherty and outphasing.
[15]	Multi-mode dual-input PA	1.2–5.7 GHz	Sigmoid power splitter	FR1/sub-7-GHz coverage; multi-mode operation	n.a.	n.a.	n.a.	Multi-mode dual-input PA with sigmoid power splitting.
[16]	Two-RF-input LM-ET PA	3.6–3.8 GHz	Load-modulated ET	Scaled/phased auxiliary RF injection	Efficiency degradation <9%	3-dB improvement at 5-dB OBO	n.a.	Two-RF-input load-modulated ET PA.
[17]	GaN characterization	mm-wave tech.	Trapping-dynamics analysis	Sub-0.15-μm GaN HEMTs; 4×50 μm periphery	Device-level peak PAE around 50%	n.a.	n.a.	Characterization of trapping and thermal effects.

**Table 2:** Numerical quantities and hyperparameters used in the customized multi-objective IWO synthesis. The reported values correspond to the final massive IWO runs used to generate the Pareto fronts and representative shaping laws.

Experimental Operating Map							
Parameter	$N_{V_{D}}$	$N_{P_{\mathrm{in}}}$	$N_{ATT}$	$N_{PH}$	$N_{\mathrm{map}}$	$K_{\max}$	
Value	33	31	22	15	337,590	≤31	
**IWO Population and Iteration Settings**							
Parameter	$N_{\mathrm{pop},0}$	$N_{\mathrm{pop},\max}$	$N_{\mathrm{it}}$	$N_{\mathrm{run}}$	$N_{\mathrm{arch}}$	$N_{\mathrm{eval}}$	
Value	80	400	160	5	1500	≤1,920,400	
**IWO Reproduction, Dispersal, and Convergence Settings**							
Parameter	$S_{\min}$	$S_{\max}$	$\sigma_{\mathrm{ini}}$	$\sigma_{\mathrm{fin}}$	$\eta_{\sigma }$	$N_{\mathrm{stall}}$	$\varepsilon_{\mathrm{stall}}$
Value	1	6	0.25	0.008	1.6	35	$10^{-5}$

**Table 3:** LM-ET special cases, the color of a cell is related to the BM reference: green for improvement, orange for degradation. The color saturation is proportional to the magnitude of the difference.

Case	$G_{\mathrm{flat}}$	Mean $P_{\mathrm{out}}$	Max $P_{\mathrm{out}}$	Mean PAE	Max PAE	Mean IMD	Max IMD	Mean OIP3	Max OIP3
ET (Benchmark)	17	33.0	37.7	0.33	0.50	31.0	38.7	18.5	19.0
BestGain	19.3	35.3	40.2	0.20	0.38	33.7	42.3	22.2	28.0
BestMeanPout	19.0	35.5	40.5	0.16	0.30	34.0	41.7	22.5	27.8
BestMeanPAE	12.4	30.0	37.0	0.45	0.53	25.2	30.7	12.6	16.3
BestMeanIMD	17.2	34.3	40.2	0.23	0.38	37.9	42.1	23.3	27.7
MidFront_75Pout_25PAE	18.0	34.8	40.4	0.24	0.36	30.7	37.8	20.2	26.2
MidFront_50Pout_50PAE	16.2	33.3	40.4	0.33	0.38	32.2	36.0	19.4	23.6
MidFront_25Pout_75PAE	14.9	31.7	39.6	0.42	0.53	28.3	33.1	15.9	21.3
MidFront_75Pout_25IMD	19.0	35.5	40.4	0.17	0.36	35.3	41.7	23.1	27.8
MidFront_50Pout_50IMD	18.9	35.1	40.2	0.20	0.38	36.8	42.3	23.5	28.0
MidFront_25Pout_75IMD	17.9	34.7	40.2	0.19	0.38	37.1	42.1	23.2	27.7
MidFront_75Pout_25Gain	19.1	35.5	40.5	0.16	0.33	34.3	41.7	22.6	27.8
MidFront_50Pout_50Gain	19.2	35.5	40.5	0.18	0.30	32.2	41.7	21.6	27.8
MidFront_25Pout_75Gain	19.3	35.4	40.5	0.19	0.34	33.4	41.7	22.1	27.8
MidFront_75PAE_25Gain	14.2	31.5	38.9	0.42	0.53	28.1	32.6	15.6	20.1
MidFront_50PAE_50Gain	16.4	33.1	40.2	0.36	0.46	30.9	36.0	18.6	24.0
MidFront_25PAE_75Gain	18.0	34.4	41.4	0.27	0.68	27.9	31.5	18.3	23.5
MidFront_75PAE_25IMD	14.2	31.6	38.9	0.41	0.49	28.7	32.6	16.0	20.1
MidFront_50PAE_50IMD	15.2	32.5	39.8	0.35	0.43	32.3	35.0	18.6	21.8
MidFront_25PAE_75IMD	16.7	33.7	40.2	0.29	0.38	35.5	38.3	21.5	24.6
MidFront_75IMD_25Gain	17.9	34.7	40.2	0.19	0.38	37.1	42.1	23.2	27.7
MidFront_50IMD_50Gain	18.8	35.1	40.2	0.20	0.38	36.8	42.3	23.5	28.0
MidFront_25IMD_75Gain	19.1	35.4	40.4	0.18	0.36	35.7	42.3	23.3	28.0
BestBalanced4D	17.3	34.2	40.4	0.25	0.36	36.4	39.4	22.4	26.0
BestKnee4D	16.7	33.5	40.2	0.32	0.39	32.8	37.0	19.9	24.5

## Data Availability

The original contributions presented in this study are included in the article. Further inquiries can be directed to the corresponding author.

## Abbreviations

The following abbreviations are used in this manuscript:

ACPR	Adjacent Channel Power Ratio
DI	Dual Input
DPD	Digital PreDistortion
EER	Envelope Elimination and Restoration
ET	Envelope Tracking
EVM	Error Vector Magnitude
HEMT	High Electron Mobility Transistor
IMD	Intermodulation Distortion
IWO	Invasive Weed Optimization
LM	Load Modulation
LM-ET	Load Modulated-Envelope Tracking
OBO	Output Back-Off
OIP3	Output Third-Order Intercept Point
PA	Power Amplifier
PAE	Power Added Efficiency
PAPR	Peak-to-Average Power Ratio
